# Supplementary material for: Identification of photoperiod-induced specific miRNAs in the adrenal glands of Sunite sheep (Ovis aries)
Source: Front Vet Sci. 2022 Jul 22;9:888207. doi: 10.3389/fvets.2022.888207 (PMC9354845; doi:10.3389/fvets.2022.888207)
Supplement: Supplementary file 1 [file Data_Sheet_1.ZIP › Supplementary file/miRDeep2 file.zip!/miRDeep2 file/Know_Structure/SP42A2/oar-mir-19b.pdf]

miRBase precursor : oar-mir-19b
Total read count : 2442
oar-miR-19b read count : 2440
remaining reads : 2

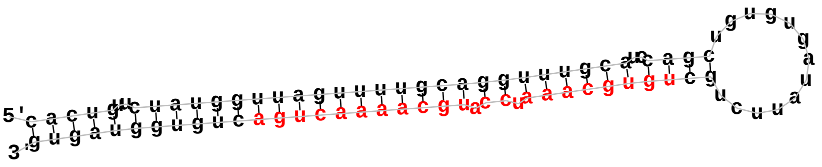

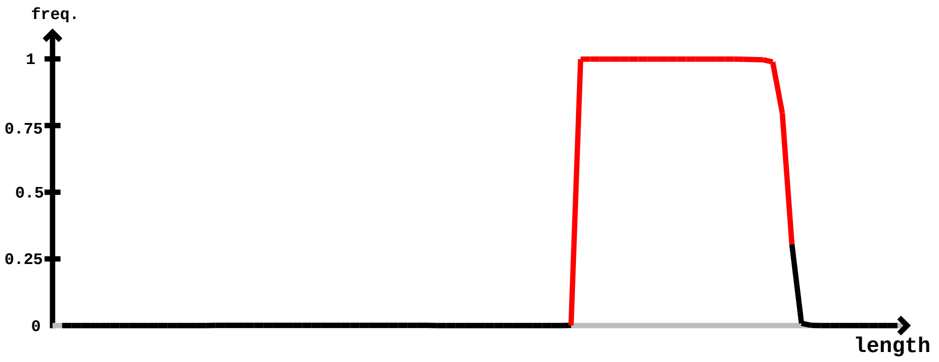

oar-miR-19b

```
5'- cacuguucuaugguuaguuuugcagguuugcauccagcugugugauauucugcugugcaaauccaugcaaaacugacugugguagug   -3'    exp
    (((((..(((((((((((((((((((((((((..((((.............)))))))))).)).))))))))))))))))))))))   reads    mm    sample
    ...............aguuuugcagguuugcauccagc.................................................   2        0     seq
    ....................................................cugugcaaauccaugcaaaacu.............   1        0     seq
    ....................................................cugugcaaauccaugcaaaacug............   1        0     seq
    .....................................................ugugcaaauccaugcaa.................   2        0     seq
    .....................................................ugugcaaauccaugcaaa................   2        0     seq
    .....................................................ugugcaaauccaugcaaaa...............   2        0     seq
    .....................................................ugugcaaaucUaugcaaaac..............   1        1     seq
    .....................................................ugugcaaauccaugcaaaac..............   19       0     seq
    .....................................................ugugcaaaucUaugcaaaacu.............   51       1     seq
    .....................................................ugugcaaauccaugcaaaacu.............   415      0     seq
    .....................................................ugugcaaauccaugcaaaacug............   1103     0     seq
    .....................................................ugugcaaaucUaugcaaaacug............   99       1     seq
    .....................................................ugugcaaauccaugcaaaacuga...........   632      0     seq
    .....................................................ugugcaaaucUaugcaaaacuga...........   90       1     seq
    .....................................................ugugcaaauccaugcaaaacugac..........   2        0     seq
    .....................................................ugugcaaauccaugcaaaacugaU..........   17       1     seq
    .....................................................ugugcaaauccaugcaaaacugaUu.........   2        1     seq
    ......................................................gugcaaauccaugcaaaacuga...........   1        0     seq
```
